# Production profile of lipid mediators in conjunctival lavage fluid in allergic and infectious conjunctivitis in guinea pigs

**DOI:** 10.3389/falgy.2023.1218447

**Published:** 2023-07-06

**Authors:** Akane Hayashi, Koji Kobayashi, Tatsuro Nakamura, Nanae Nagata, Takahisa Murata

**Affiliations:** ^1^ Animal Radiology, Graduate School of Agricultural and Life Sciences, The University of Tokyo, Tokyo, Japan; ^2^ Food and Animal Systemics, Graduate School of Agricultural and Life Sciences, The University of Tokyo, Tokyo, Japan; ^3^ Veterinary Pharmacology, Graduate School of Agricultural and Life Sciences, The University of Tokyo, Tokyo, Japan

**Keywords:** conjunctivitis, lipid mediators, tear, biomarker, eosinophils, neutrophils

## Abstract

**Introduction:**

Conjunctivitis is a major ocular disease classified into allergic or infectious. The pathological features of conjunctivitis are not fully understood despite its high morbidity rate; thus, its differentiation can be difficult.

**Materials and methods:**

We used ovalbumin-induced allergic conjunctivitis and lipopolysaccharide-induced infectious conjunctivitis models of guinea pigs. Both models showed conjunctival swelling. Histological studies revealed that numerous eosinophils infiltrated the conjunctiva in the allergic model, whereas neutrophils infiltrated the conjunctiva in the infectious model. We collected conjunctival lavage fluid (COLF) and comprehensively analyzed lipid production using liquid chromatography-tandem mass spectrometry.

**Results:**

COLF showed increase of 20 and 12 lipid species levels in the allergic and infectious models, respectively. Specifically, the levels of a major allergic mediator, prostaglandin D_2_ and its three metabolites and several cytochrome P450-catalyzed lipids increased in the allergic model. In the infectious model, the levels of prostaglandin E_2_ and 8-iso-prostaglandin E_2_ increased, indicating tissue inflammation. Moreover, the level of 12-oxo-eicosatetraenoic acid, a lipoxygenase metabolite, increased in the infectious model.

**Conclusion:**

These differences in lipid production in the COLF reflected the pathological features of allergic and infectious conjunctivitis.

## 1. Introduction

Conjunctivitis is one of the most common ocular diseases. It induces several symptoms including conjunctival itching, redness, edema, and discharge. It is classified into allergic and infectious conjunctivitis (AC and IC, respectively) according to its pathogenesis (1). AC is caused by the hypersensitivity to aeroallergens, such as pollen and animal dander. Eosinophils play an important role in its pathogenesis (2). IC is caused by microbial infections, mostly by viruses or bacteria. Neutrophil invasion of the conjunctiva is its main pathological feature (3). Despite the high morbidity rates, the pathological features of conjunctivitis are not fully understood.

AC and IC are diagnosed based on patients' history and clinical signs. However, the distinction between AC and IC is often ambiguous (1), leading to a misdiagnosis. General practitioners' diagnoses of AC and IC are not sufficiently accurate, with positive predictive values of 67% for AC and 71% for IC (4). Incorrect and delayed diagnosis leads to an inappropriate use of antibiotics or corticosteroids, as well as the recurrence and/or mass morbidity of IC, particularly among pediatric patients. Therefore, novel diagnostic methods with high sensitivities and specificities are required.

Lipid mediators are locally produced bioactive molecules that regulate various physiological and pathophysiological processes including inflammation (5). They are derived from polyunsaturated fatty acids (PUFAs) such as arachidonic acid (AA), eicosapentaenoic acid (EPA), and linoleic acid (LA). These PUFAs are metabolized into lipid mediators by enzymes such as cyclooxygenase (COX), lipoxygenase (LOX), and cytochrome P450 (CYP) or by non-enzymatic oxidation. Recent developments in liquid chromatography-tandem mass spectrometry (LC-MS/MS) have enabled a sensitive and comprehensive analysis of these bioactive lipid mediators.

Since the lipid production profile varies according to the type and stage of the disease, it is useful in investigating disease progression and identifying disease biomarkers. In particular, several types of secretory and excretory samples, including nasal mucus, tears, and urine, have received attention because of their easy and non-invasive sampling process. These samples are easy to apply clinically, and their non-invasiveness allows us to investigate the body's inflammatory status more accurately. For example, by assessing the lipid profile in nasal lavage fluid in mouse allergic rhinitis model, we previously found that the level of 12-hydroxyeicosatetraenoic acid (12-HETE) was elevated in allergic rhinitis, and it promoted late-phase responses of the disease (6). Moreover, we identified tetranor-prostaglandin (PG) D metabolite (PGDM) as a novel index for food allergy symptoms by profiling urinary lipids in patients with food allergy (7). Furthermore, we found that urinary levels of PGE_2_ and PGF_2α_ metabolites increased in a murine model and in patients with atopic dermatitis (8). These studies have clearly shown that the lipid profile assessment of these biological samples is useful in identifying new therapeutic targets and biomarkers.

Several studies have suggested that lipid mediators are produced during conjunctivitis and that they play important roles in disease progression (9, 10). For example, the levels of several lipids, including PGD_2_, PGF_2α_, and leukotriene (LT) B_4_ increased in the mice's conjunctiva in an AC model induced by short ragweed pollen (9). Ocular infection with *Pseudomonas aeruginosa*, a bacterial IC, increased the levels of PGE_2_, LTB_4_, and thromboxane (TX) B_2_ in the mice's ocular tissue (10). These facts suggest that the lipid profiles in AC and IC are distinct. Therefore, we can understand each pathological feature by comparing them.

Experimental allergy models in various types of animals such as rats and mice have been used to study allergic reactions in humans. Guinea pig models have been frequently used because histamine and leukotrienes are involved similar to human pathology (11). In this study, we comprehensively analyzed the production of 158 lipid species in the conjunctival lavage fluid (COLF) of AC and IC guinea pigs using LC-MS/MS and identified distinct lipid profiles in AC and IC that reflected disease pathology.

## 2. Materials and methods

### 2.1. Animals

Hartley guinea pigs (6-weeks old, male) were purchased from Shiraishi animals Co., Inc. (Saitama, Japan). They were housed under 12-h dark/light cycle and given *ad libitum* access to water and feed. All the experiments were approved by the Institutional Animal Care and Use Committee of the University of Tokyo (P18-039, P23-006).

### 2.2. Allergic conjunctivitis model

**Figure 1:**
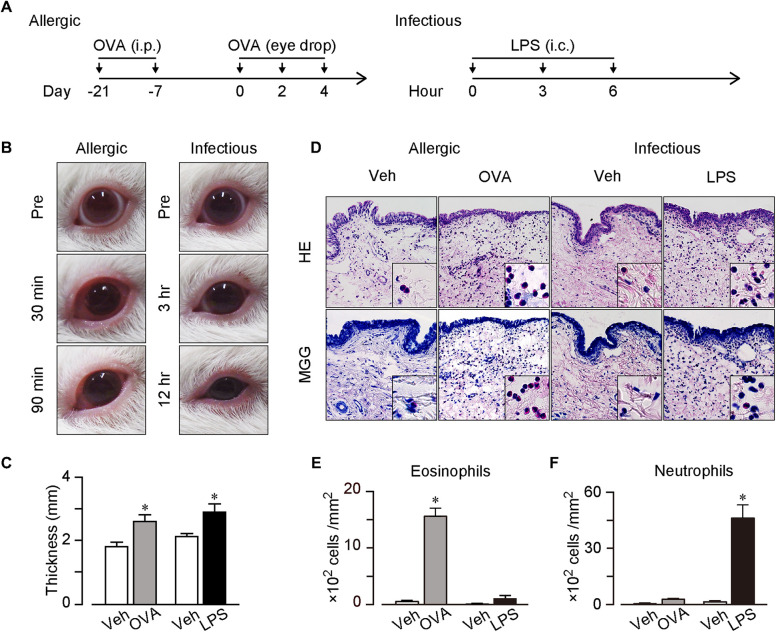
AC and IC models. (**A**) Protocols for the models. For AC model, guinea pigs were sensitized twice (on day −21 and −7) by intraperitoneal injection of 100 μg of OVA and 1 mg of Alum. For stimulation, 50 µg of OVA in 10 μl saline was dropped onto the left conjunctival sac three times (on day 0, 2 and 4). For IC model, guinea pigs received intraconjunctival injection of 5 μg of LPS to the upper and lower left conjunctiva three times (at 0, 3 and 6 h). (**B**) Typical pictures of OVA or LPS-treated eyes. Left panels; before (pre), and 30 and 90 min after the last OVA treatment. Right panels; before (pre), and 3 and 12 h after the first LPS treatment. (**C**) Thickness of the upper conjunctiva. (**D**) Typical images of hematoxylin & eosin (H&E) stained sections and May-Grunwald Giemsa (MGG) stained sections. (**E,F**) Number of neutrophils (**E**) and eosinophils (**F**) in mucosal surface of conjunctiva per mm^2^ (*n* = 4 each). Data are presented as mean ± SEM. *P < 0.05.

Guinea pigs were sensitized twice (on day −21 and −7) by intraperitoneal injection of 100 μg of ovalbumin (OVA; Sigma-Aldrich, St. Louis, MO) and 1 mg of aluminum potassium sulfate (Alum; Sigma-Aldrich) in 200 μl saline (Figure 1A). For stimulation, 50 µg of OVA in 10 μl saline was dropped onto the left conjunctival sac three times (on day 0, 2 and 4) under isoflurane anesthesia. The same amount of saline was dropped onto the right conjunctival sac as the control. For pathological and histological analysis, guinea pigs were euthanized under pentobarbital and conjunctival tissue was dissected 90 min after the last stimulation.

### 2.3. Infectious conjunctivitis model

Guinea pigs received intraconjunctival injection of 5 μg of lipopolysaccharide (LPS; Sigma-Aldrich) in 50 μl saline to the upper and lower left conjunctiva three times (at 0, 3 and 6 h) under isoflurane anesthesia (Figure 1A). The same amount of saline was injected to the right conjunctiva as the control. For pathological and histological analysis, conjunctival tissue was dissected 6 h after the last stimulation.

### 2.4. Isolation of conjunctival lavage fluid

Conjunctival lavage fluid (COLF) was collected by washing the conjunctival sac gently with 20 μl saline twice under isoflurane anesthesia. The sample collection was performed 30 min after the last OVA stimulation or 6 h after the last LPS stimulation. COLF of untreated guinea pigs were also collected as naïve sample. Collected samples were stored at −80°C until use for lipid measurement.

### 2.5. Evaluation of edema

Thickness of the dissected upper palpebral conjunctiva was measured using caliper immediately after resection.

### 2.6. Histopathological analysis

Palpebral conjunctivae were excised from the guinea pigs, immediately immersed in 4% paraformaldehyde (4°C, overnight) and embedded in paraffin. Sections (4 μm) were stained with hematoxylin & eosin (H&E) or May-Grunwald stain solution and Giemsa stain solution (May-Grunwald Giemsa staining, MGG) by the conventional methods. Stained sections were pictured using BZ-X700 microscope (Keyence, Kyoto, Japan). We counted neutrophils or eosinophils in 100 μm squares from randomly selected 10 fields in the mucosal surface for each section to calculate the mean infiltrated cells. The results were shown as cells per mm^2^.

### 2.7. Measurement of lipids

COLF samples (40 µl) were mixed with distilled water (185 µl), 0.05% formic acid (225 µl), methanol (100 µl) and internal standard (IS) solution (50 µl, shown in Supplementary Table S1). The mixed solutions were loaded onto methanol- and water-conditioned solid-phase extraction cartridge (MonoSpin C18, GL Sciences, Tokyo, Japan). The cartridge was washed with water (300 µl) and hexane (300 µl) and then the lipids were eluted by loading methanol (50 µl) twice. 5 µl sample solution was injected to LC-MS/MS (LCMS-8060, Shimadzu, Kyoto, Japan) and measured with LC-MS/MS Method Package for Lipid Mediators (ver.2) as manufacturers instruction. In short, liquid chromatographic separation was performed by using Kinetex C8 column (2.1 mm × 150 mm, 2.0 µm, Phenomenex, Torrance, CA, USA) and the mobile phase with the liner gradient step shown in Supplementary Table S2.

### 2.8. Data processing

The data were shown as mean ± SEM. The amounts of lipids were calculated with the peak area of chromatogram in each lipid and normalized by the IS substances. The data were shown as the ratio to IS, i.e., intensity of objective component/intensity of internal IS substance in each sample. Statistical evaluation for the symptom data was performed by one-way ANOVA followed by Dunnett's test. For lipids data, Kruskal-Wallis test followed by Steel's test for comparison was used. A value of *P *< 0.05 was taken as significant.

## 3. Results

### 3.1. AC and IC guinea pig models

Regarding the AC model, we sensitized guinea pigs twice with an intraperitoneal OVA injection (100 µg, day −21 and −7) and administered OVA into the conjunctival sac thrice at days 0, 2, and 4 (Figure 1A). After the last OVA administration, the guinea pigs exhibited swelling and edema in both the palpebral and bulbar conjunctivae within 10 min. The symptoms were the most severe 30 min after the OVA administration and continued for at least 90 min (Figure 1B). Regarding the IC model, we injected LPS (5 µg) intraconjunctivally thrice at a 3 h interval (Figure 1A). The LPS injection induced swelling in both the palpebral and bulbar conjunctivae, and the symptoms worsened with time (Figure 1B). The guinea pigs' eyes narrowed 6 and 12 h after the first LPS injection.

Conjunctival tissues were dissected 90 min after the last OVA administration and 12 h after the first LPS injection. The conjunctival thickness increased in both the OVA-induced AC and LPS-induced IC models (0.8-mm increase in the AC model and 1.1-mm increase in the IC model, Figure 1C).

### 3.2. Histological analysis

We performed histological analysis of the conjunctival tissues and observed a few inflammatory cells in the vehicle-treated conjunctiva, whereas numbers of segmented granulocytes were observed in both OVA- and LPS-treated conjunctivae in the H&E-stained sections (Figure 1D). High-power field images showed that the OVA treatment induced an eosinophil infiltration, whereas the LPS treatment induced a neutrophil infiltration (Figure 1D). Furthermore, we confirmed the eosinophil infiltration in the OVA-treated conjunctiva via MGG staining, which stained eosinophils red (Figure 1D). As shown in Figure 1E, the OVA treatment increased the number of eosinophils (30-folds increase), whereas the LPS treatment did not (8-folds increase). In contrast, the OVA treatment did not significantly increase the number of neutrophils (5-folds increase), whereas the LPS treatment did (34-folds increase, Figure 1F). Therefore, we successfully established two conjunctivitis models that mimicked human conjunctivitis: an AC model with an eosinophilic inflammation and an IC model with a neutrophilic inflammation.

### 3.3. Arachidonic acid-metabolites in COLF in the AC and IC models

**Figure 2:**
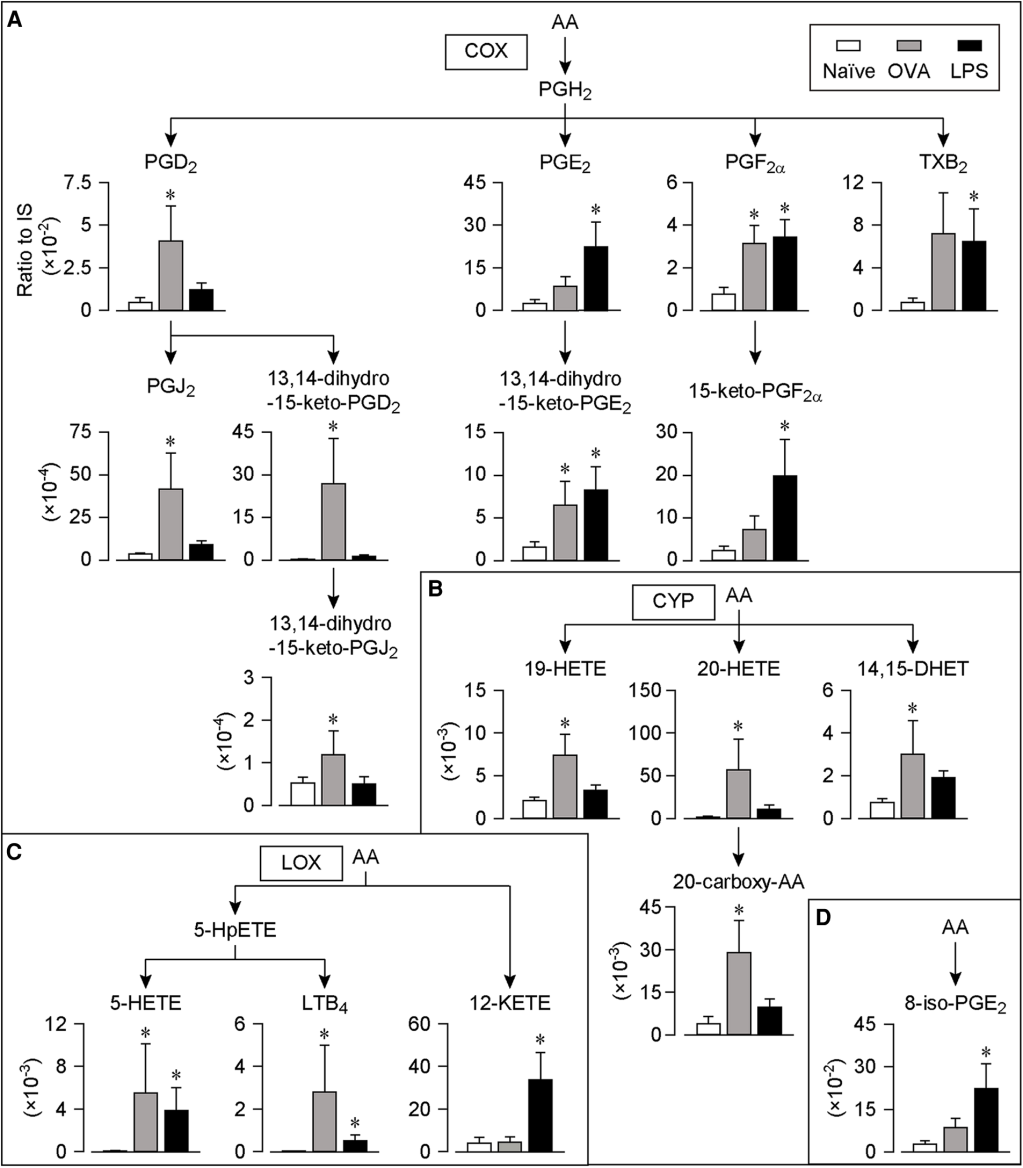
AA-derived lipids in COLF of AC and IC models. (**A–D**) Relative amount of each lipid of AC and IC. The lipids were arranged according to the metabolic pathways. Ratio of the peak chromatogram area of objective lipid to that of IS were shown. COX-catalyzed lipids (**A**), CYP-catalyzed lipids (**B**), LOX-catalyzed lipids (**C**), and lipids catalyzed by non-enzymatic oxidation (**D**). White bar; naïve, Gray bar; AC model, Black bar; IC model, *n* = 7 each. Data are presented as mean ± SEM. *P < 0.05. AA, arachidonic acid; COX, cyclooxygenase; CYP, cytochrome P450; LOX, lipoxygenase; PG, prostaglandin; TX, thromboxane; HETE, hydroxy-eicosatetraenoic acid; DHET, dihydroxyeicosatrienoic acid; KETE, oxo-eicosatetraenoic acid; LT, leukotriene.

**Figure 3:**
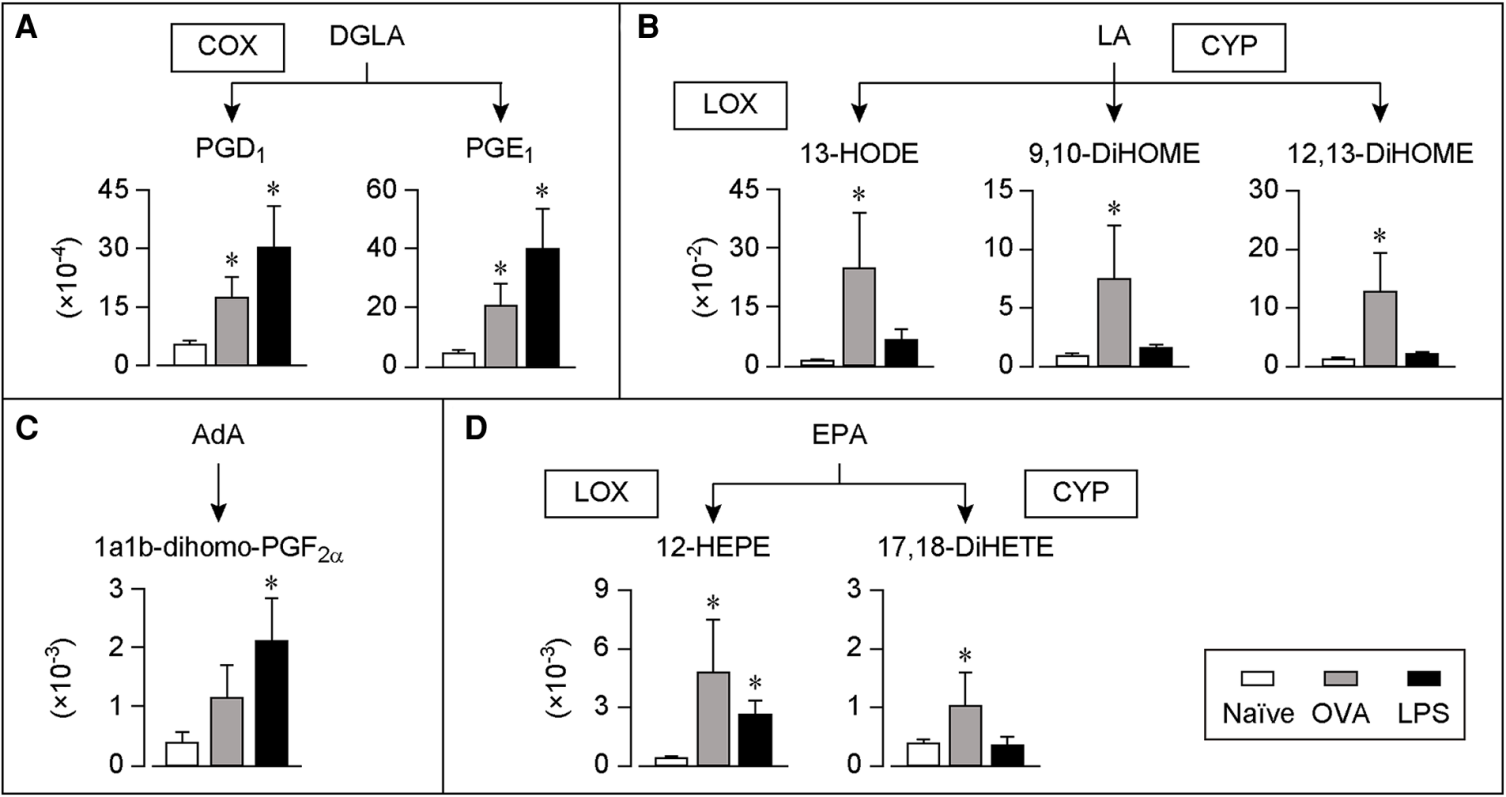
DGLA, LA, AdA or EPA-derived lipids in COLF of AC and IC models. (**A–D**) Relative amount of each lipid of AC and IC. The lipids were arranged according to the metabolic pathways. Ratio of the peak chromatogram area of objective lipid to that of IS is shown. DGLA-derived lipids (**A**), LA-derived lipids (**B**), AdA-derived lipids (**C**), and EPA-derived lipids (**D**). White bar; naïve, Gray bar; AC model, Black bar; IC model, *n* = 7 each. Data are presented as mean ± SEM. *P < 0.05. COX, cyclooxygenase; LOX, lipoxygenase; CYP, cytochrome P450; DGLA, dihomo-gamma-linolenic acid; EPA, eicosapentaenoic acid; LA, linoleic acid; AdA, adrenic acid; PG, prostaglandin; DiHETE, dihydroxy-eicosatetraenoic acid; HODE, hydroxy-octadecadienoic acid; DiHOME, dihydroxy-octadecenoic acid; HEPE, hydroxy-eicosapentaenoic acid.

We collected COLF by washing the conjunctival sac 30 min after the last OVA stimulation or 6 h after the last LPS stimulation and performed a comprehensive analysis of lipids using LC-MS/MS. These time points were selected because the symptoms including edema and redness of the conjunctivae were the most severe according to the appearance. We found 25 lipid species whose levels were significantly increased in both the AC and IC models. These lipids were mapped according to their metabolic pathways (Figures 2, 3).

Figure 2 summarizes the production of AA metabolites. In the AC model, the levels of a major allergic mediator PGD_2_ and its metabolites, PGJ_2_, 13,14-dihydro-15-keto-PGD_2_, and 13,14-dihydro-15-keto-PGJ_2_ consistently increased (Figure 2A). Moreover, the levels of the PGE_2_ metabolites 13,14-dihydro-15-keto-PGE_2_ and PGF_2α_ increased. The level of TXB_2_ tended to increase in the AC model, but this increase was not statistically significant. In the IC model, the levels of PGD_2_ and its metabolites did not change, whereas those of the major pro-inflammatory mediators, PGE_2_, PGF_2α_, TXB_2_, and their metabolites (13,14-dihydro-15-keto-PGE_2_ and 15-keto-PGF_2α_) increased.

As shown in Figure 2B, the levels of four CYP-catalyzed lipids from AA, 19-HETE, 20-HETE, its metabolite, 20-carboxy-AA, and 14,15- dihydroxy-eicosatrienoic acid (DHET) increased only in the AC model. Moreover, the levels of the LOX-catalyzed lipids 5-HETE and LTB_4_ increased in the AC model (Figure 2C). The levels of 5-HETE, LTB_4_, and 12-oxo-eicosatetraenoic acid (KETE) increased in the IC model. Furthermore, the levels of 8-iso-PGE_2_ produced by the non-enzymatic oxidation of AA increased only in the IC model (Figure 2D).

### 3.4. Other lipid metabolites in COLF in the AC and IC models

Figures 3A–C summarizes the production of lipids derived from the AA precursors, dihomo-gamma-linolenic acid (DGLA) and LA, or its metabolite, adrenic acid (AdA). The levels of the two COX-catalyzed lipids from DGLA, PGD_1_, and PGE_1_, increased in both the AC and IC models (Figure 3A). Furthermore, the levels of the LA-derived lipids, LOX-catalyzed 13-hydroxy-octadecadienoic acid (HODE) and CYP-catalyzed 9,10- or 12,13-dihydroxy-octadecenoic acid (DiHOME) increased in the AC model, whereas their levels did not change in the IC model (Figure 3B). In contrast, the levels of the AdA-derived lipid, 1a1b-dihomo-PGF_2α_, increased only in the IC model (Figure 3C).

Figure 3D summarizes the production of lipids derived from EPA, which is a precursor of anti-inflammatory lipid mediators (12). The levels of a LOX-catalyzed lipid, 12-hydroxy-eicosapentaenoic acid (HEPE) and a CYP-catalyzed lipid, 17,18-dihydroxy-eicosatetraenoic acid (DiHETE), increased in the AC model. Moreover, the levels of 12-HEPE increased in the IC model.

## 4. Discussion

We used comparable guinea pig models of AC and IC that exhibited similar symptoms and found different production profiles of lipid mediators in COLF. In the AC model, PGD_2_ and its metabolites, 13-HODE, and several CYP-catalyzed lipids predominantly increased. In contrast, in the IC model, PGE_2_, 8-iso-PGE_2_, and 12-KETE increased. PGF_2α_, PGD_1_, PGE_1_, 5-HETE, and LTB_4_ increased in both the AC and IC models. Detailed results and information regarding each lipid are summarized in Table 1.

**Table 1:** Summary list of the detected lipids, its cellular sources and functions.

Increased in	Lipid	Enzyme	Possible sources	Previous reports	References
Allergic	PGD_2_	COX	Eosinophils, mast cells	▪ function as an autocrine signal for eosinophil activation and chemotaxis (human, *in vitro*) ▪ aggravate allergic diseases such as rhinosinusitis, conjunctivitis, and asthma (mouse or guinea pig, *in vivo*)	(13, 14)
	13-HODE	15-LOX	Eosinophils	▪ promote allergic airway inflammation (mouse, *in vivo*)	(15, 16)
	19-HETE	CYP	Unknown	few reports	
	20-HETE			▪ induce airway inflammation and increase Th2 inflammation-related gene expression (mouse, *in vivo*)	(17)
	9,10-DiHOME			few reports	
	12,13-DiHOME			▪ suppress the differentiation of regulatory T cells (human, *in vitro*) ▪ might heighten the risk of developing asthma in infant (human)	(18)
	17,18-DiHETE (EpETE*)			▪ suppress inflammation in several models such as food allergy and skin inflammation (mouse, *in vivo*)	(19, 20)
Infectious	PGE_2_	COX	Neutrophils, macrophages, various cells	▪ promote neutrophil recruitment in several skin inflammation models (mouse, *in vivo*) ▪ inhibit excessive neutrophil accumulation and activation in infectious model (mouse, *in vivo*)	(21–25)
	TXB(A*)_2_			▪ aggravate acute lung inflammation by increasing vascular hyper-permeability (mouse, *in vivo*)	(26)
	12-KETE	12-LOX	Unknown	few reports	
	8-iso-PGE_2_	OX^a^	Neutrophils	▪ might promote neutrophil infiltration via increasing neutrophil adhesion and endothelial cell permeability (human and pig, *in vitro*)	(27, 28)
	1a,1b-dihomo-PGF_2α_	COX/OX	Neutrophils, macrophages	few reports	
Both	PGF_2α_	COX	Various cells	▪ aggravate LPS-induced systemic inflammation through anti-inflammatory cytokine production (mouse, *in vivo*)	(29)
	PGD_1_			▪ might promote Th2 cell and eosinophil infiltration through crth2 activation (*in vitro*)	(30)
	PGE_1_			▪ enhance histamine-induced infiltration of inflammatory cells into the conjunctiva (rabbit, *in vivo*)	(31)
	5-HETE	5-LOX	Leukocytes	▪ promote leukocyte infiltration (human and several species, both *in vivo* and *in vitro*)	(32–35)
	LTB_4_				
	12-HEPE	12-LOX	Unknown	▪ suppress skin inflammation (mouse, *in vivo*)	(36)

The functions of the original lipids (*) are listed for several lipids.

^a^ OX, non-enzymatic oxidation.

Hirakata et al. showed that the levels of several lipids, including PGD_2_, PGF_2α_, and LTB_4_, increased in the conjunctival tissue in a mouse AC model (9). These observations are consistent with those of the present study using COLF. Ambaw et al. investigated the lipid profiles of patients with meibomian gland dysfunction and found that several lipids, including 5-HETE, LTB_4_ and 18-HEPE, can be useful indices for disease severity (37). Thus, lipids in tears or COLF can reflect the tissue inflammatory status and can be useful indices for determining the state of eye diseases.

The AC model exhibited a severe eosinophil accumulation in the conjunctiva, which is the main pathological feature of AC in humans (2). A previous study has shown that eosinophil-derived PGD_2_ functioned as an autocrine signal for eosinophil activation and chemotaxis (13). This autocrine pathway likely promoted AC pathogenesis. Eosinophils also produced 13-HODE (15). Mabalirajan et al. reported that intranasal injection of 13-HODE induced airway inflammation and that its neutralization decreased allergic airway inflammation in a mouse model (16), suggesting its pro-allergic role in AC. Furthermore, some CYP-catalyzed lipids produced in the AC model have been involved in allergic diseases. For example, an intra-tracheal injection of 20-HETE induced airway inflammation and increased the mRNA expression of several Th2 inflammation-related genes, including IL-13 (17). The administration of 12,13-DiHOME suppressed the differentiation of regulatory T-cells, and its increase in feces during infancy increased the risk of asthma (18). These lipids can be novel therapeutic targets for treating AC.

The IC model exhibited a severe neutrophil accumulation in the conjunctival tissue, which is a typical feature of bacterial IC in humans (3). Activated neutrophils produce PGE_2_ (21), indicating that neutrophils may be a source of PGE_2_ in the IC model. PGE_2_ exerted both pro- and anti-inflammatory effects depending on the type and stage of inflammation. The administration of PGE_2_ promoted neutrophil recruitment in several mouse models (22, 23). Other studies have demonstrated that PGE_2_ inhibition aggravated inflammation through excessive neutrophil accumulation and activation (24, 25). Further studies are required to elucidate the role of PGE_2_ in IC. Activated neutrophils produce reactive oxygen species that can produce 8-iso-PGE_2_ via the non-enzymatic oxidation of AA. 8-iso-PGE_2_ stimulates neutrophil inflammation by increasing neutrophil adhesion (27) and endothelial cell permeability (28). The sources and functions of 12-KETE are poorly understood. Further investigation of these factors will provide new insights.

In both the AC and IC models, the levels of several COX-catalyzed lipids increased. COXs are expressed in various cell types and their expression and activation are upregulated during tissue damage. Therefore, the increases in both the AC and IC models were reasonable. Topical treatment with PGE_1_ enhanced the histamine-induced infiltration of inflammatory cells into the rabbit conjunctiva (31), suggesting that these lipids play a role in disease pathogenesis. Eosinophils and neutrophils highly express 5-LOX (32), and its products 5-HETE and LTB_4_ are major chemo-attractants for these cells (33–35). These lipids may function as autocrine signaling molecules for leukocyte chemotaxis to promote AC and IC.

In the present study, we did not examine the changes in lipid production profile according to each disease progression, and did not elucidate the detail mechanisms underlying the difference between AC and IC. Further studies are needed to elucidate these points.

In conclusion, we have found different lipid production profiles in the COLF in AC and IC. We considered that this difference was derived from the distinct pathological features of AC and IC. Our findings have provided new insights into the disease pathology and potential diagnostic markers for AC and IC.

## Data Availability

The original contributions presented in the study are included in the article/**Supplementary Material**, further inquiries can be directed to the corresponding author.
