# Supplementary material for: Production profile of lipid mediators in conjunctival lavage fluid in allergic and infectious conjunctivitis in guinea pigs
Source: Front Allergy. 2023 Jul 6;4:1218447. doi: 10.3389/falgy.2023.1218447 (PMC10358838; doi:10.3389/falgy.2023.1218447)
Supplement: Supplementary file 1 [file Table1.docx]

**Supplementary Table 1** The composition of internal standards mixture for comprehensive analysis

| Substance | | Concentration |
| --- | --- | --- |
|  |  | (ng/mL in ethanol) |
| tetranor-PGEM-d_6_ | 200 | |
| 6-keto-PGF_1α_-d_4_ | 2,000 | |
| TXB_2_-d_4_ | 200 | |
| PGF_2α_-d_4_ | 200 | |
| PGE_2_-d_4_ | 200 | |
| PGD_2_-d_4_ | 200 | |
| LTC_4_-d_5_ | 200 | |
| LTB_4_-d_4_ | 200 | |
| 5(S)-HETE-d_8_ | 1,000 | |
| 12(S)-HETE-d_8_ | 500 | |
| 15(S)-HETE-d_8_ | 200 | |
| PAF C16-d_4_ | 200 | |
| OEA-d_4_ | 40 | |

**Supplementary Table 2** Gradient program for LC-MS/MS analysis

| Step | Time (min) | Mobile Phase A (%) | Mobile Phase B (%) |
| --- | --- | --- | --- |
| 0 | 0 | 90 | 10 |
| 1 | 5 | 75 | 25 |
| 2 | 10 | 65 | 35 |
| 3 | 20 | 25 | 75 |
| 4 | 25 | 5 | 95 |
| 5 | 27 | 90 | 10 |
